# Influence of Two Different Cryoextraction Procedures on the Quality of Wine Produced from Muscat Grapes

**DOI:** 10.3390/foods9111529

**Published:** 2020-10-24

**Authors:** Ana Ruiz-Rodríguez, Enrique Durán-Guerrero, Ramón Natera, Miguel Palma, Carmelo G. Barroso

**Affiliations:** Department of Analytical Chemistry, Center for Agri-Food and Wine Research (IVAGRO), Faculty of Sciences, University of Cadiz, 11510 Puerto Real, Spain; ana.ruiz@uca.es (A.R.-R.); enrique.duranguerrero@uca.es (E.D.-G.); ramon.natera@uca.es (R.N.); carmelo.garcia@uca.es (C.G.B.)

**Keywords:** grapes, wines, cryoextraction, volatile compounds

## Abstract

Freezing grapes is a winemaking technique known as cryoextraction that intends to modify the composition of the final wines. The changes that take place in the frozen grapes facilitate the transfer of certain compounds from the grape skins into the musts because of the grape’s unstructured tissues. For this study, the white grape variety Muscat of Alexandria was selected. Two different cryoextraction procedures have been analyzed as follows: (i) Ultrafast freezing, and (ii) liquid nitrogen freezing. The wines obtained using liquid nitrogen freezing exhibited higher levels of terpenoids, as well as higher levels of hydroxylic compounds and fatty acids than both the wines obtained through traditional methods and ultrafast freezing wines. In any case, both freezing techniques produced wines of a more intense aroma compared with those wines obtained by traditional methods. In fact, liquid nitrogen freezing produced the wines with the most intense aroma and were the best valued by the tasting panel.

## 1. Introduction

Numerous studies have been reported in the literature dealing with the phenomena involved in the extraction of aroma components from grape skins into grape juice during the preferential maceration of the juice and skin from white grapes [1,2,3]. In particular, and in the case of Muscat of Alexandria, the white grape discussed here, it has been found that the contact between grape juice and skin enhances the aromas of the variety and the resulting wines are notable for their mint and melissa aroma notes [4,5,6].

As a modification of the classical prefermentative maceration at low temperature, different techniques have been developed that involve freezing the grapes; a method known as cryoextraction [7]. Freezing the grapes does not necessarily affect their organoleptic qualities [8,9]. When the grapes are frozen, ice crystals are formed and these tear the pectocellulose walls, thus disorganizing their tissues and facilitating skin compound extraction processes. In fact, at a small-scale the freezing of grapes did not cause any adverse effects with regard to their vinification and facilitated a more rapid extraction of their aroma compounds [10]. In some cases, two different techniques were applied: Firstly, cryoextraction (freezing the grape prior to its pressing) and, secondly, supraextraction (freezing the grape followed by defrosting and pressing). It is clear that for both techniques the grapes need to be at least partially defrosted, although the difference lies with the time lapse before starting the pressing procedures, and not with the time required to freeze the grapes. Either of these two techniques gives place to fresher, more structured and pleasant wines with a long persistence [11]. Some interesting examples of wine production that involves grape freezing have been found, including the freezing of Syrah grapes by means of solid carbon dioxide that intends to provide wines with a more intense color [12], or the freezing of Nebbiolo grapes to produce more aromatic wines after their proper fermentation [13].

The freezing speed of the grapes is a particularly important aspect to be taken into account, since it is directly related to the degree of disorganization of the berrys’ structure [14,15,16]. Casassa and Sari [17] studied the effect of external refrigeration by means of solid carbon dioxide on Malbec grapes and they discovered that more intensely colored wines were obtained when pellets of solid carbon dioxide were employed. Both solid carbon dioxide (−78.5 °C) and liquid nitrogen (−195.8 °C) induce a thermal shock that leads to a greater degradation of the berries, which in turn prolongs the contact between pulp and must, with the additional benefit of protecting the grapes from the effect of oxygen [18]. In contrast, the use of other external refrigeration methods—such as refrigeration chambers (between −18 °C and −28 °C)—even if less aggressive, does not isolate the macerating must from oxygen. On the other hand, chamber freezing allows a closer control of the process, so that the freezing of tissues can be avoided to reduce the level of bitter tannins transferred into the wine and thus favor the balance of the final product [19].

The effect of the different cryogenic techniques can be more easily evaluated in those grape varieties with specific compounds of interest in their skin. One such example is provided by the variety Muscat of Alexandria, where the level of the varietal characteristic aroma are particularly high in their berrys’ skin. In fact, this grape variety has been frequently used to evaluate the effect of different cultivation conditions as well as winemaking techniques on the resulting wines [20]. Some such studies include the analysis of molecular aspects, while others have mainly focused on the effect of grape maturity and the development stages of its skin and pulp [21,22] on the final wine characteristic including the peppermint aroma [23]. In all of these studies, high levels of terpenes, such as linalool, geraniol, nerol and citronerol, were found to provide successful results.

The work described here intends to determine the influence that cryoextraction may have on the elaboration of white wine from Muscat of Alexandria grapes. Two freezing methods have been evaluated and these have been related to their specific freezing rates. The final objective is to determine the effect that each one of these two freezing procedures may have on the wines obtained and particularly with regard to their content in aroma related and phenolic components as well as on their actual sensory qualities.

## 2. Materials and Methods

### 2.1. Winemaking

**Figure 1:**
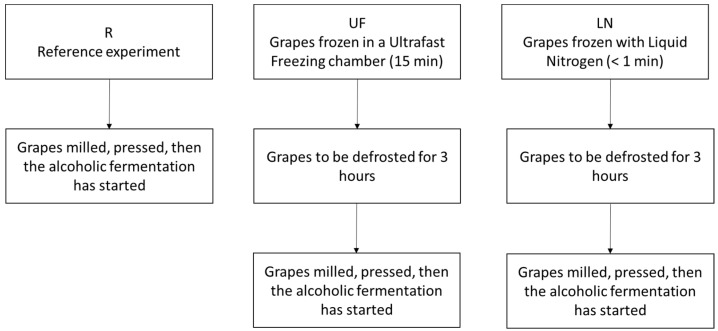
Winemaking processes for the three types of wine: R (reference), UF (ultra fast mechanical freezing) and LN (liquid nitrogen freezing).

Muscat of Alexandria grapes grown in the area of Jerez de la Frontera (Cádiz, Spain) were used to produce white wine. Three different vinification procedures were implemented (Figure 1).

Approximately 900 kg (100 kg × 3 replicates × 3 type of wines) of grapes from this variety were used to produce the wines. Three different tanks were used for each wine type. The first wine was the “reference” (R) sample, whose grapes had been crushed at room temperature (20 °C) and then pressed. The second wine was denoted as “ultra fast mechanical freezing” (UF). The grapes that were used to produce this second wine had been deep-frozen in a highly powerful freezing chamber that would bring the grapes down to −28 °C in 15 min. The third wine was labeled as “liquid nitrogen freezing” (LN) and the grapes used to produce this third wine type were frozen for less than 1 min by means of liquid nitrogen. The grapes frozen by either technique were stored in the same chamber at −18 °C for 2 h. The bunches were then allowed to thaw at room temperature for 3 h. The semi-thawed grapes were then crushed and pressed.

The must for each winemaking process was corrected to a pH of 3.3 using tartaric acid and 40 mg L^−1^ sulfur dioxide was also added as potassium metabisulfite. The yeast *Saccharomycescerevisiae* var. *bayanus*, commercially known as “viniferm PDM” commercialized by (Agrovin, Ciudad Real, Spain) was used for the fermentation process in 35 liter containers. The fermentation started 24 h after the inoculation of the yeast and carried on for 11 days, never exceeding 20 °C, until the end of the process when the reducing sugars reached below 5 g L^−1^. The wine from each winemaking process was cold stabilized in a refrigerator at 4 °C. Sulfur dioxide was added to 80 mg L^−1^ of total sulfur dioxide and it was filtered through 0.4–0.6 micron filtering plates.

### 2.2. Characterization of Musts and Wines

Both grape juices and wines were characterized by determining three routine parameters (sugar content, pH and total acidity) as well as total polyphenols. All the determinations were carried out in triplicate. The density measurements were performed by means of an Anton Paar DMA 4500M electronic densimeter (Graz, Austria) in order to determine the sugar levels in the musts based on their direct correlation with density. It was also used to determine ethanol level in 200 mL wine distillate samples. The acidity was calculated by acid-base titration using a Crison-Hach model pHmatic 23 (Dusseldorf, Germany) automatic titrator by adding NaOH 0.1 M until pH = 7.0. It was expressed as g L^−1^ of tartaric acid.

The polyphenol content of the wines was determined according to the protocol by Mazza et al. after a minor modification [24]. Briefly, in order to measure the total polyphenols in the samples, their absorbance was registered at 280 nm using a Jasco V-530 UV/VIS spectrophotometer. Seven standard solutions (80–1000 mg L^−1^ gallic acid) were used to express the result as gallic acid equivalents. For most wines and must, the results from this method correlated with the results from the Folin-Ciocalteau method [25].

### 2.3. Determination of Aroma Compounds

The stir bar sorptive extraction (SBSE) technique was used in conjunction with GC-MS to determine volatile components content of each wine. The extractions were carried out using 10 mm × 0.5 mm (length × film thickness) PDMS commercial stir bars, supplied by Gerstel (Mulheim a/d Ruhr, Germany). Each sample was run in duplicate and the average values of the three types of wine corresponding to each winemaking processes were used for later discussions. A volume of 25 mL of each wine type was pipetted and placed into a 100-mL Erlenmeyer flask containing 5.85 g of NaCl and 50 L of a solution formed by 4-methyl-2-pentanol (2.27 g L^−1^ in Milli-Q water containing 5 g L^−1^ of tartaric acid). The Erlenmeyer flask was placed on a 15-samples magnetic stirrer. The stir bar was stirred at 1250 rpm at 25 °C for 120 min. Then, the stir bar was removed from the wine sample and submerged for a few seconds into distilled water in order to remove any NaCl and subsequently gently wiped dry using a lint-free tissue. It was then transferred into a glass thermal desorption tube to be thermally desorbed by means of a commercial TDU thermal desorption unit (Gerstel) connected to a programmed-temperature vaporisation (PTV) injector CIS-4(Gerstel) through a heated transfer line. The PTV was installed on an Agilent 6890 GC-5973 MS system (Agilent Technologies, Palo Alto, CA, USA). An empty baffled liner was used with the PTV. The thermodesorption unit was equipped with a MultiPurpose Sampler (MPS) 2 L autosampler (Gerstel) with capacity for 98 coated stir bars. The desorption temperature was programmed from 40 to 300 °C (held for 10 min) at 60 °C min^−1^ under a helium flow (75 mL min^−1^) and the desorbed analytes were cryofocused using the PTV system with liquid nitrogen at −140 °C. Finally, the PTV system was programmed from −140 to 300 °C (held for 5 min) at 10 °C s^−1^ for analysis by GC–MS. The capillary GC–MS analyses in the electron impact mode were performed on an Agilent 6890 GC-5973N MS system (Agilent, Little Falls, DE, USA), equipped with a DB-Wax capillary column (J&W Scientific, Folsom, CA, USA), 60 m × 0.25 mm I.D., with a 0.25 micron coating. Helium was used as the carrier gas at a flow rate of 1.1 mL min^−1^. The detector temperature was 250 °C. The GC oven was programmed as follows: Held at 35 °C for 10 min, then ramped at 5 °C min^−1^ up to 100 °C. Then it was raised to 210 °C at 3 °C min^−1^ and held for 40 min. The peak identifications were carried out based on the Wiley library by analogy with mass spectra and this was confirmed by pattern retention indices, whenever possible, or according to the retention data reported in the literature. The quantitative data for the identified compounds were obtained by measuring the relative area of the molecular ion peak of each compound relative to that of 4-methyl-2-pentanol, the internal standard [26]. The relative standard deviation values using this method ranged from 3.27% (nerol) to 8.73% (4-vinylguaiacol) for terpenoids, from 2.19% (isoamyl alcohol) to 9.03% (1-hexanol) for hydroxylic compounds, from 2.54% (benzaldehyde) to 7.38 (nonanal) for aldehydes, from 1.36% (octanoic acid) to 11.06% (tetradecanoid acid) for fatty acids and from 3.64% (isoamyl acetate) to 7.40% (ethyl hexadecanoate) for ethyl esters.

### 2.4. Tasting Methodology

The sessions were carried out in a normalized tasting room (UNE-EN ISO 8589:2010) so that any influences from external stimuli on the judgments would be minimized. A panel formed by twelve members who were considered as experts took part in the tasting sessions. The panel members were either winemakers or equally experienced laboratory staff members selected for their consistent assessments.

The wine samples from the 3 replicates of each specific winemaking process were blended together before starting the sensory analysis. The sessions consisted of presenting the three types of wines to the judges for comparative purposes. Exactly 50 µL of wine was poured into a tasting glass (UNE-EN ISO 3591:1977) fitted with a lid to minimize aroma losses. Each glass was identified by a 3-digit code. The room temperature was set at 22 °C and the samples were presented randomly.

The sensory analysis of the wines was carried out in two sessions. In the first session, the judges were requested to perform an ordering test (UNE-EN ISO 8587:2010) to determine their preferences. This type of test is classified as discriminative and it is especially applicable when there are several samples to be compared, since it minimizes the consumption of the samples and the sensory fatigue of the judges. After presenting the wine samples before the judges, they were asked to evaluate and order them according to their preferences (from lowest to highest preference). The wine ID codes were noted down on the corresponding forms. Each judge was encouraged to include additional comments at his/her discretion, since such comments could contribute to a deeper interpretation of the quantitative data.

In the second session, the judges were asked to grant a score on the impact notes for each wine sample according to a 1 to 5 scale. The specific impact notes were selected from the conclusions of the first session.

### 2.5. Statistical Analysis

Wines were prepared in triplicate, i.e., three different fermentation tanks were used for each grape juice and analyses were done in triplicate. An analysis of variance (ANOVA) was performed on the continuous variables, i.e., sugar, total acidity, pH, total polyphenols and volatile compounds and the Xi-square test was applied to the discrete variable, i.e., the sensory panel results. The differences were considered as relevant when the *p*-value was less than 0.05. The statistical analyses were carried out by means of an SPSS V27 (IBM, Armonk, NY, USA).

## 3. Results and Discussion

### 3.1. Characteristics of the Resulting Musts

**Figure 2:**
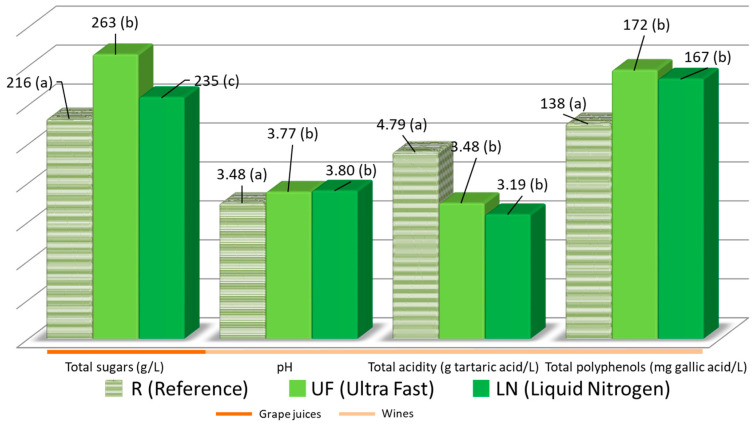
Average levels or the routine parameters in the different grape juices and wines (R, UF and LN). Different letters in brackets indicate a significant difference (*p*-value < 0.05) for each parameter.

The grapes were frozen, thawed and then pressed. The resulting musts were analyzed in order to ascertain whether there were differences in their properties that could be attributed to the different treatments applied to the grapes. The results for total sugars, pH and total acidity in the musts are shown in Figure 2.

Regarding the total acidity of the musts, it should be mentioned that the reference must, which was obtained without submitting its grape to any specific treatment, had the highest value (4.79 ± 0.43 vs. 3.48 ± 0.17 and 3.19 ± 0.21 g of tartaric acid L^−1^). This result is a consequence of the precipitation of potassium salts during the freezing process and the lower solubility of the acidic salts when the grapes were defrosted—both effects had already been reported in the literature [27]. This process is also responsible for the different pH levels between the three musts. The highest pH values were related to the musts obtained after freezing/defrosting the grapes. No significant differences were found between the wines obtained using frozen grapes.

Regarding their sugar levels, the reference must (R) showed the lowest sugar content (216 ± 5) when compared to the musts obtained after freezing the grapes (263 ± 4 (UF) and 235 ± 4 (LN)) (Figure 2). One of the peculiarities of cryoextraction is that on pressing partially defrosted grapes, part of their juice defrosts faster because of a cryoscopic decrease of the melting point that takes place in the berry areas where the concentration of sugar is higher. Significant differences were found among the three grape musts.

Different grape freezing techniques have different effects on the final wines. When the grapes are frozen by means of liquid nitrogen (LN) the wines that are obtained have a greater total sugar content than those obtained when the grapes are frozen by ultra fast mechanical freezing (UF). The difference in sugar content might be a result of a faster defrosting process in the case of ultra fast mechanical freezing, while when grapes have been frozen by means of liquid nitrogen the effect of cryoscopic decrease would be more evident and, consequently, the musts obtained should exhibit higher sugar contents. In an effort to avoid the enzymatic oxidation of the grapes during the defrosting process, the grinding process was started just three hours after the defrosting of the grapes had begun. At that point, the thawing of the grapes was more advanced in those grapes that had undergone ultra fast mechanical freezing when compared to those which had been frozen by Liquid Nitrogen.

The alcoholic level of the R wine was 11.2 ± 0.21, 15.2 ± 0.36 for the LN wine, and 13.4 ± 0.41 for the UF wine. These values reflect the differences in must sugar content initial values and underline the first major effect caused by the two freezing techniques used.

### 3.2. Total Polyphenols in the Wines

Cryoextraction should produce wine with higher levels of polyphenols as they are easily extracted from the grape skins [12]. However, longer treatments before the alcoholic fermentation can promote the oxidation of polyphenols [28]. In order to identify the predominant effect, i.e., either greater extraction of polyphenols or greater oxidation of the total phenolic components, both effects were determined or the resulting values are presented in Figure 2.

It was confirmed that the wines from frozen grapes presented higher phenolic contents with no significant differences between them (138 ± 11 for the R wine, 172 ± 8 for the LN wine and 167 ± 9 for the UF wine expressed as mg L^−1^ of gallic acid). No significant differences were found between the two wines obtained from frozen grapes (LN and UF). For this reason, it can be assumed that during the short freezing process, the freezing procedure does not matter, the grapes are degraded to a sufficient extent so that more polyphenols are extracted from their skin during the grinding and pressing process than in the case of berries that had not been subjected to freezing at all, even if both grinding and pressing are processes that are carried out at lower temperatures. This effect has been previously described in the literature although for longer freezing times [29,30]. If this is the case, a similar trend should be found with regard to those other compounds that originate in grape skin and that give the wine its particular aroma. Other aspects, such as solubility level may also be a relevant factor with regard to the prevalence of polyphenol extraction or phenolic oxidation.

### 3.3. Individual Aroma Components in the Wines

The aromas of Muscat of Alexandria wines were studied by analyzing the concentrations of terpenes, alcohols, aldehydes, fatty acids and ethyl esters and significant differences (*p*-value < 0.05) were found between the different winemaking techniques used in this study. Most of these compounds are present in grape skins, therefore, any differences between the wines from frozen grapes should be attributed to differences in the degradation level of the grapes skin over the freezing process. It has been previously described that freezing processes may affect some volatile compounds such as the thiols in Sauvignon blanc grapes [31], however, in those cases the effects were due to longer freezing periods that resulted in certain biochemical changes. In our study, a very short freezing period was applied in every case and, therefore, only mechanical changes in the grapes skin would explain the differences in the final composition of the wines.

**Figure 3:**
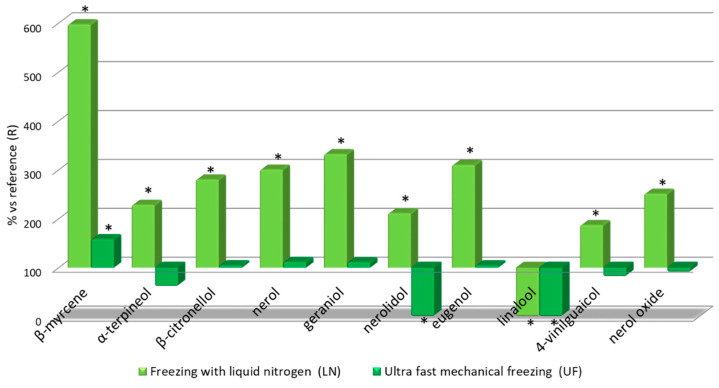
Relative levels of volatile terpenoids in the three wine types: Relative values for LN and UF vs R. * Significant difference vs the R wines (*p*-value < 0.05).

**Figure 4:**
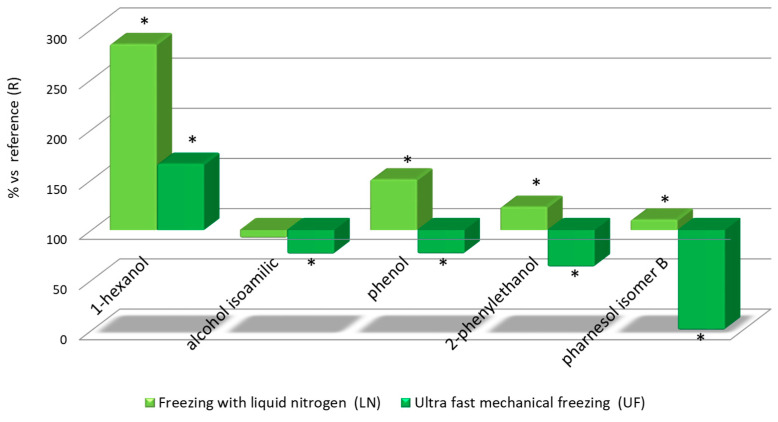
Relative levels of volatile alcohols in the three wine types: Relative values for LN and UF vs R. *Significant difference vs the R wines (*p*-value < 0.05).

**Figure 5:**
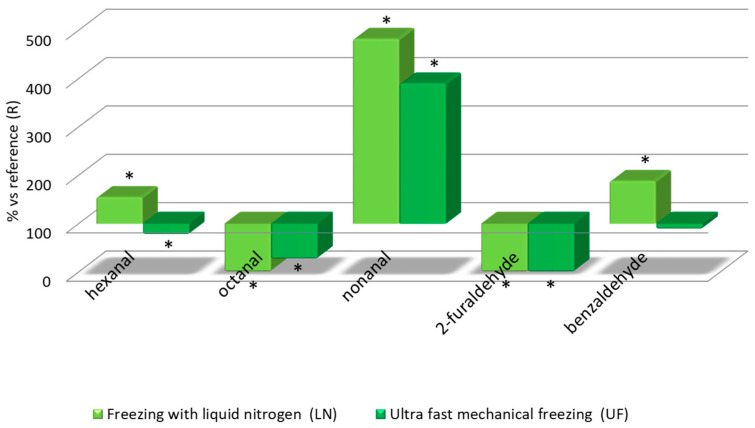
Relative levels of volatile aldehydes in the three wine types: Relative values for LN and UF vs R. * Significant difference vs. the R wines (*p*-value < 0.05).

**Figure 6:**
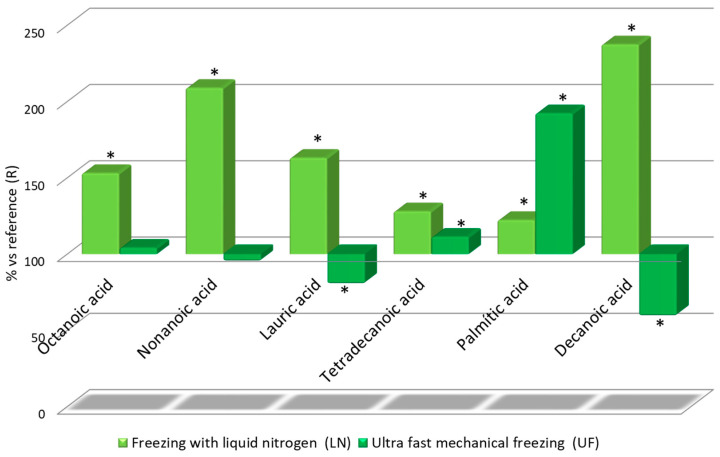
Relative levels of volatile fatty acids in the three wine types: Relative values for LN and UF vs R. * Significant difference vs. the R wines (*p*-value < 0.05).

**Figure 7:**
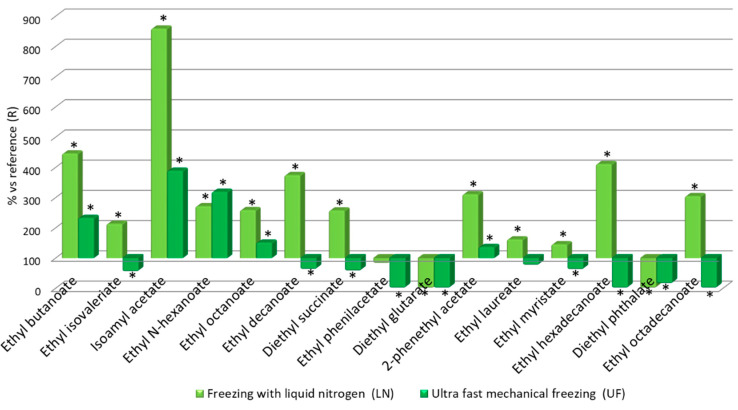
Relative levels of volatile ethyl esters in the three wine types: Relative values for LN and UF vs R. * Significant difference vs. the R wines (*p*-value < 0.05).

Given the large number of the parameters analyzed related to wine aroma, out of convenience, only their average relative values were represented to be compared against the reference wine (R), which was given a value of 100%. The results are presented in Figure 3 Figure 4 Figure 5 Figure 6 Figure 7.

It can be seen in Figure 3 that several of the characteristic components of the varietal aroma that were analyzed exhibited higher values in the two wines from frozen grapes than those found in the reference wine (R) and, in many cases, such values were as much as three fold the ones in non-frozen grape wines. The effects of freezing techniques on terpenoids have been previously found also for other grape varieties [32]. These results are consistent with the previously mentioned skin degradation that occurs during freezing, which facilitates the transfer of skin origin components into the must. The above outlined results also seem to indicate that the net degradation of these components does not occur during the freezing and handling of the grapes in the manner previously described. The LN wine had a significantly higher concentration of terpenes than the reference wine, while the UF wine only exhibited slightly and hardly relevant higher values than the reference wine. The above observation is specified in percentage terms as follows: All the terpenes in the LN wine reached a value greater than 150% compared to wine R, while in the UF wine only β-myrcene exceeded that value (158%) with respect to wine R, only linalool showed a lower value in both LN and UF wines. These compounds are responsible for the aroma balsamic notes of wines [33], therefore freezing processes will increase the balsamic notes in Muscat wines.

β-myrcene content in the LN wine was almost 600% that in the reference wine (R), whereas nerol (301%), geraniol (332%) and eugenol (310%) exhibited three fold levels compared to the R wine. It is also worth highlighting other components that differed from those in the reference wine by a factor of two or more. For example, in the LN wine these components include terpineol (229%), although linalool was present at a lower level than in the R wine. Terpineol is related to mint aroma [34] and nerol oxide (238%) is related to flower aroma [33]. In the UF wine, only β-myrcene was present at higher levels than in the reference wine. Therefore, if we take into account the contribution of these compounds to the specific aroma of Muscat wines, grape freezing techniques seem to have a positive effect and such effect is clearly noticeable in the LN wines. However, it must be noted that no new compounds were detected on LN nor UF wines vs the R wines.

The data obtained from the determination of alcohol contents confirm that the LN wines always presented higher concentrations than the UF wines followed by the R wine. It is worth mentioning that, as can be seen in Figure 4, the difference in 1-hexanol content reached a significant 250% in the LN wines when compared to the reference wine. The UF wines also presented a marked difference with respect to the reference wine with a 50% increment. Similar results have been previously obtained in other investigations on the same and other grape varieties [35]. In this case, freezing would negatively affect the final wines in comparison to winemaking without freezing. 1-Hexanol provides herbaceous notes to wine aroma because of the enzymatic oxidation of the fatty acids in the grapes. This process is related to the breakage of the grain and the processes to which it is subjected from its harvesting until the beginning of the alcoholic fermentation. The LN wines had also higher concentrations of the other alcohols than both the reference and the UF wines. These differences were in most cases more evident when compared to the R wine, in any case, much lower than the differences found for 1-hexanol. Additionally, those alcohols are less important than 1-hexanol for the wines aroma properties. (Figure 4).

Regarding the analysis of aldehydes, their behavior is more heterogeneous with respect to the different techniques used in the winemaking process. Nonanal is the component with the most noticeable concentration difference with respect to the R wine: The LN wine reached a value of 482% and the UF wine went as high as 390%. Nonanal has been associated to citrus fruits aromas [36]. The LN wines also contained hexanal and benzaldehyde at 50% higher levels than the reference wine. These compounds are responsible for burnt sugar and almond related flavors in wines [33,36]. On the other hand, octanal and 2-furaldehyde had lower concentrations in the frozen grape wines than in the R wine but the differences were not so relevant (Figure 5). Although their final contribution to the aroma of wine is not well understood, aldehydes that have 8–10 carbon atoms are considered to be strong odorants. These compounds include (E)-2-nonenal, octanal, nonanal, decanal and (E,Z)-2,6-nonadienal [34]. Regarding the rest of the aldehydes, they have not been thoroughly understood and may give either pleasant or unpleasant aroma notes [37]. Therefore, because of the higher levels of compounds related to the citrus fruits aroma, the LN and UF wines will have more interesting aroma properties.

The study of fatty acids produced similar results to those corresponding to terpene-like components. The LN wines exhibited higher values for fatty acids than the UF wines. Their content in octanoic acid (153%), nonanoic acid (208%), lauric acid (163%) and decanoic acid (207%) were over that in the R wine by even more than 50%. A similar trend was detected in the UF wines for palmitic acid, with 190% its content in the reference wine, which is even higher than the levels found in the LN wines (Figure 6). It is again noteworthy that there were some cases where the content levels more than double those in the reference wine, such as nonanoic acid and decanoic acid in the LN wines. The UF wines, however, did not show such a marked increment with respect to the R wine.

Fermentation conditions can affect the composition of the fatty acids in a particular wine. Under anaerobic conditions, yeast produces medium-chain fatty acids and when fermentation is carried out in aerobic or semi-aerobic conditions more unsaturated fatty acids are produced [38]. However, the fermentation conditions for the three tests completed in our study were the same. Therefore, the only factor that could affect the composition of the initial fatty acids in the musts would be the treatment applied to the grapes (pressing, maceration or clarification), which in turn would affect the final composition of the wines.

Ethyl esters are formed because of the esterification of fatty acids during the alcoholic fermentation of must. The acetates from higher alcohols and the ethyl esters from fatty acids are associated to floral and fruity aromas in young wines [39]. Therefore, they are usually appreciated in young wines. Linear (C2–C4), medium (C6–C10), long (C6–C10) and branched (2-methyl propanoic, 2-methyl butanoic, etc.) volatile fatty acids are produced during fermentation, and it has been proven that as the length of their chains increase, their volatility decreases and wines’ odor changes from acid to rancid [40]. The LN wines presented higher concentrations of these various components compared with the UF wines. The LN wines showed increments greater than 200% with respect to the reference wine for the following components: Ethyl butanoate (446%), ethyl isovalerate (213%), isoamyl acetate (858%), ethyl n-hexanoate (271%), ethyl octanoate (258%), ethyl decanoate (374%), diethyl succinate (257%), 2-phenylethyl acetate (311%), ethyl hexadecanoate (411%) and ethyl octadecanoate (305%), whereas their content levels in the UF wine were: Ethyl butanoate (232%), isoamyl acetate (389%), and ethyl n-caproate (319%) (Figure 7). The larger amounts of these compounds determined in the LN wine could be attributed to a greater presence of non-esterified organic acids.

### 3.4. Tasting Rating

**Figure 8:**
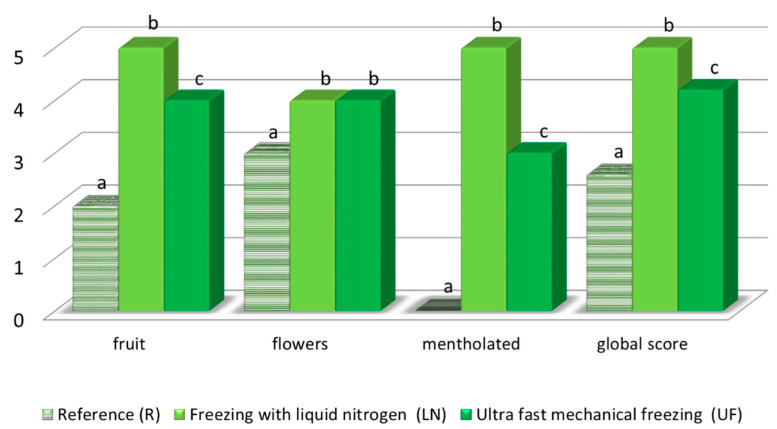
Panel tasting results for R, LN and UF wines. Different letters indicate a significant difference (*p*-value < 0.05) for each parameter.

On tasting the wines, all the members in the panel considered the reference wine to be the least aromatic of all the three wine types. The LN and UF wines were given significantly higher scores for fruit and flower notes with respect to the R wine that had be produced by traditional methods (Figure 8). This result is consistent with the analysis of the individual aroma components, such as ethyl butanoate, ethyl n-hexanoate, ethyl octaoate, ethyl decanoate, diethyl succinate and ethyl myristate, which were described as fruit flavors [33] and that had been found predominantly in the UF and LN wines. Likewise, nonanal, which provides citrus notes [36], was a major component in the UF and LN wines. While, 2-phenylethyl acetate and nerol oxide, usually associated to floral notes, was found in large concentrations in the LN wines [33].

The panelists were able to differentiate the LN from the UF wines by its remarkable menthol notes, which made the former wine unique and attractive. The compounds responsible for these notes seem to be monoterpenes and their derivatives [41,42]. β-Myrcene has been said to confer balsamic notes [33] while terpineol provides mint notes [34]; both of these compounds were determined at higher concentrations in the LN wines compared to the other two wines.

Regarding the overall rating (Figure 8), the LN wines reached the highest score, while the reference wine was the least valued one. Significant global score differences were noticeable between the reference wine against both LN and UF wines. However the differences between the two wines that had been produced from frozen grapes were not so considerable. This overall rating is in agreement with the above mentioned specific aroma and flavor characteristics exhibited by each wine type.

## 4. Conclusions

Freezing procedures reduced acidity, increased alcoholic strength and, above all, produced wines that were more aromatic, since they contain greater amounts of the components that are more closely associated to Muscat grape wine specific characteristics.

The wines produced using liquid nitrogen to freeze the grapes presented higher concentrations of terpenes in all the cases, whereas the wines produced using the ultrafast freezing chamber exhibited similar although just slightly higher values when compared to the reference wine. β-citronerol, nerol and geraniol contents were particularly high in the wines from frozen grapes using liquid nitrogen, with more than double their concentration with respect to the control wine. These results were consistent with the score granted by the tasting panel who judged them as characteristic wines of special interest.

With regard to the difference between wines produced by the two freezing techniques applied in this study, liquid nitrogen frozen grape wines, not only contained more of the compounds that provide Muscat wines with their characteristic fruity and mentholated notes, but they were also more appreciated by the judging panelist than those wines obtained from fast frozen grapes.
